# Texas’ “One Man” Health Department

**Published:** 1895-01

**Authors:** 


### EDITORIAL DEPARTMENT.

F. E. DANIEL, M. D., Editor.
S. E. HUDSON, M. D., Managing Editor.
A. J. SMITH. M. D., Galveston, Associate Editor.

EDITORIAL STAFF:

PROF. J. E. THOMPSON, M. D., Texas Medical College, Galveston; Surgery.
PROF. WM. KEILLER, M. D., Texas Medical College, Galveston; Obstetrics and Gynecology.
PROF. DAVID CERNA, M. D., Texas Medical College, Galveston; Therapeutics.
PROF. A. J. SMITH, M. D., Texas Medical College, Galveston; Medicine
DR. ISADORE DYER, Tulane University; Dermatology.
DR. R. H. L. BIBB, Saltillo, Mexico; Foreign Correspondent.

Official organ of the West Texas Medical Association, the Houston District Medical Association, the Austin District Medical Society, the Galveston County Medical Society, and several others.

### EDITORIAL.

TEXAS' "ONE MAN" HEALTH DEPARTMENT.—*How is it?* Texas has no Board of Health, and less money is allowed for the protection of the public health than perhaps in any State in the Union, and less is expended. Even the very small appropriation of $45,000 a year for all quarantine and sanitary purposes is not all used, but the total expenses have, for several years, been kept safely within the appropriation, notwithstanding a large part of it has gone for building and repairs of stations,—something which in other States is given a separate appropriation.

The health system in operation in Texas, devised as it now exists, and put in operation by the present State Health Officer (he is the author of the bill enacted into a law by the 22d legislature, which created the office of County Physician, and instituted co-operation between county and State for the suppression of local epidemics, whereby a share of the expense —all of which formerly fell on the State, now is borne by the county), has been derided as a "one man quarantine" by certain envious or disappointed parties who deny that Texas has any health system at all. Yet,—and yet,—how is it that Texas is, and for so long has been free from any serious epidemic disease, notwithstanding the frequent appearance of deadly infection at times at various points in the State, and that, too, simultaneous outbreaks of small-pox, diphtheria, scarlet fever, at points hundred of miles apart? Has any serious, or rather, extensive epidemic yet resulted? Have not several cases of scarlet fever suddenly appeared at Temple, for instance,—or of diphtheria at Waco,—or, as very recently, in a most malignant form, at Hondo, near San Antonio; and have they not been promptly extinguished, like a small blaze, and *not allowed* to spread?

Four years ago, before the present law was enacted, before the present head of the department was reappointed, * the small-pox, introduced across the Rio Grande from our Mexican neighbors with whom it is perennial, endemic, and amongst the more indigent and ignorant, cultivated you may say—-for, Dr. Yandell, health officer of El Paso, says they will purposely expose their children to the infection, that they may have the disease and be done with it, die or be protected through life,—broke out in local epidemic form in forty-six places in Texas. That is, upon accession to office, the present head of the health department had forty-six local epidemics of small-pox to deal with; there were in all, some where near two thousand cases, but no very extensive epidemic in any one locality occurred, unless we except San Antonio. Within a reasonably short time the State was free of the disease, and with the exception of a trifling number of cases within the last two years, has remained so, and is to-day entirely free from small-pox. And yet, to put down these forty-six outbreaks, entailed little expense upon the State, the little appropriation for quarantine purposes not being even used up. How is it, then, that in Texas, notwithstanding her "one man concern," epidemics do not get a start? Whereas, in Ohio, for instance, as at present in Cleveland, there are hundreds of cases of scarlet fever; and in other States and cities we read of the spread and ravages of diphtheria? In Columbus, Ohio, the scarlet fever made its appearance in the public schools, and despite their boards of health, and the boasted advanced sanitation, and every facility for enforcing it, the disease spread, the schools are now closed, and all the doctors in the city in consultation as to how to stop it. In Chicago recently, small-pox became alarmingly epidemic, (and we know that Illinois has one of the most efficient health organizations in the world),—the number of cases mounting up into the thousands.

* Dr. S. was State Health Officer from 1880 to 1894 inclusive, except 1889 and 1890.

Elsewhere in this issue, we publish a circular letter, issued by the State Health Officer, the head of our wonderful "one-horse," no—"one man" health system, which may throw some light upon the "why." It is because, the first case is *isolated*, and all who have been exposed are quarantined till after the danger period; and all the necessary precautions against a spread are taken at the inception; the first *spark* is extinguished, and a conflagration is prevented. It is easier to prevent a fire than to extinguish one. Our "one man" seems, if he is but one man, to be ubiquitous. The first news the *people* have of an outbreak of dangerous disease anywhere, is the news that the State Health Officer is *upon the ground*, and that the resources of *modern preventive medicine* have been invoked to prevent a spread.

Have not the advantages of the Texas system been demonstrated within the past few years,—and that, too, without the expenditure of even so much as $45,000 a year? The appropriation which will be asked for '95, will be only $42,000. The State Health Officer feels assured, that without the occurrence of something now unforeseen, he will be able to run the one-horse concern on even less than heretofore; and $2,000 of that asked for will be to provide for bacteriological examinations. On this point we will have something to say in our next. The good results of which we are boasting, and with cause,—the JOURNAL thinks we have reason to be proud of the showing—the remarkable exemption from epidemic disease—is still more remarkable from the fact that the Texas health authorities have few of the facilities placed at the disposal of health boards in other States. We should, by all means, be equipped with facilities for bacteriological research for diagnostic purposes, and also with facilities for the new diphtheria treatment; but more of this, later. As to yellow fever, our people have learned to rest in peaceful security. Infected vessels have several times arrived at Galveston quarantine, but—"thus far and no farther";—the days of yellow fever epidemics in Texas are, we hope, happily over.

